# Regression of vascular calcification following an acute episode of calciphylaxis: a case report

**DOI:** 10.1186/1752-1947-8-52

**Published:** 2014-02-14

**Authors:** Hui-Tsung Yeh, Ing-Jer Huang, Chien-Ming Chen, Yao-Min Hung

**Affiliations:** 1 Hong Yi Clinic of Nephrology, 309 MinZu Road, Chiayi City 600, Taiwan; 2 Department of Plastic and Reconstructive Surgery, Chiayi Branch, Chang Gung Memorial Hospital, 6 West Section, Jiapu Road, Puzi City, Chiayi County 61363, Taiwan; 3 Department of Emergency Medicine, Kaohsiung Veterans General Hospital, 386 Dazhong 1st Road, Zuoying District, Kaohsiung City 81362, Taiwan

**Keywords:** Calciphylaxis, Cinacalcet, Sodium thiosulfate, Vascular calcification

## Abstract

**Introduction:**

In clinical situations, vascular calcification tends to progress and is difficult to completely arrest or reverse. Calciphylaxis, a severe complication of end-stage renal disease, is a specific form of vascular calcification. Control studies have provided evidence that monotherapy with sodium thiosulfate or cinacalcet delays the progression of vascular calcification. Successful treatment of calciphylaxis with sodium thiosulfate or cinacalcet has also been reported. We report a case demonstrating the regression of vascular calcification following an acute episode of necrotic skin lesions suspected to be calciphylaxis. During the successful multimodal treatment, sodium thiosulfate and cinacalcet were sequentially administered in addition to surgical debridement and percutaneous transluminal angioplasty.

**Case presentation:**

We describe the case of a 71-year-old Asian woman on hemodialysis who presented with suspected calciphylaxis lesions in her lower left leg. Plain radiographs revealed diffuse calcified vessel changes in her lower extremities. During the initial wound treatment with a course of intravenous sodium thiosulfate, our patient’s predialysis serum levels of total calcium markedly increased, yielding no calciphylaxis improvement. The necrotic wounds began healing only after surgical debridement. A percutaneous transluminal angioplasty was performed to dilate a 70% stenosis in her left posterior tibial artery. Our patient was then treated with cinacalcet, resulting in improved control of her calcium, phosphate and parathyroid hormone serum levels. The lesions completely healed after six months of multimodal treatment. Repeated plain radiographs in the following two years revealed gradual vascular calcification regression in her lower extremities.

**Conclusion:**

In addition to the favorable outcome of our patient’s wounds, radiology was used to document the regression of calcification in the large and small arteries of her lower limbs. However, it is difficult to determine the precise mechanism of the multimodal treatment that caused the vascular calcification regression and wound healing. The clinical course suggested that the surgical treatment and percutaneous transluminal angioplasty substantially contributed to healing her wounds. Cinacalcet and sodium thiosulfate may have played distinct roles in the regression of her vascular calcification. A well-controlled study or large case series are required to assess the additive effects of these agents when treating vascular calcification.

## Introduction

In addition to accelerated atherosclerosis, patients with end-stage renal disease are subject to diffuse medial calcification in the blood vessels; this is associated with increased cardiovascular mortality. Calciphylaxis, also known as calcific uremic arteriolopathy, is a specific and devastating form of vascular calcification (VC) and represents a severe complication of end-stage renal disease. Calciphylaxis is characterized by calcification in the media of the small- to medium-sized blood vessels of the dermis and subcutaneous fat, with or without endovascular fibrosis, causing thrombotic occlusion and resultant ischemic ulcers and tissue necrosis. Caucasian ethnicity, female sex, obesity, hyperphosphatemia, hypercalcemia and the use of warfarin are known risk factors. Early recognition and prompt treatment are vital, and clinical suspicion is a critical feature of the diagnosis. Research has indicated that positive pathologic findings, such as arteriolar calcification, subintimal fibroplasia, thrombosis and ischemic cutaneous necrosis, are the standard of diagnosis. However, skin biopsy procedures are subject to sampling errors and can initiate ulcer formation and propagation [1]. A bone scan can reveal abnormal subcutaneous uptake in clinically apparent areas of disease, but this is likely only true regarding non-ulcerating lesions in viable tissue or deep and widespread lesions. Plain radiographs of the relevant soft tissues can demonstrate calcification among small blood vessels and play a role in diagnostics [1-3]. Treating calciphylaxis is challenging and multidisciplinary because of the lack of an optimal medical therapy; it involves strictly controlling the mineral metabolism, avoiding calcium and vitamin D analogues, and conducting diligent wound care, including scrupulous surgical management. Certain studies have reported successful treatment of calciphylaxis with sodium thiosulfate (STS) or cinacalcet [4]. In clinical situations, established VC tends to progress and is difficult to completely arrest or reverse. Recent studies have focused on how STS or cinacalcet affect the evolution of VC, but further research is necessary to elucidate the optimal use of these agents.

## Case presentation

A 71-year-old Asian woman with end-stage renal disease, who had undergone eight years of hemodialysis, presented with spontaneous painful plaque in her left lower calf with the surrounding skin exhibiting violaceous discoloration. Our patient had hypertension, dyslipidemia and osteoporosis but no history of diabetes, connective tissue diseases or chronic viral hepatitis. Because of recurrent thrombosis at the vascular access point for hemodialysis, she had received six months of warfarin therapy that ended five months prior to developing the calf lesion. Our patient had a long history of secondary hyperparathyroidism and had been treated with intravenous calcitriol for many years. Because of frequent episodes of hypercalcemia and hyperphosphatemia, intravenous calcitriol had to be discontinued so often as to result in inadequate control of her secondary hyperparathyroidism. Despite replacing the calcium-containing phosphate binders with sevelamer and shifting calcitriol to paricalcitol, her metabolic problems with calcium and phosphorous showed little improvement. In the previous four years, her serum levels of intact parathyroid hormone typically ranged from 350 to 800pg/mL, her calcium levels were around 9.5 to 10.6mg/dL and her phosphate serum levels were approximately 5.0 to 7.4mg/dL.

**Figure 1:**
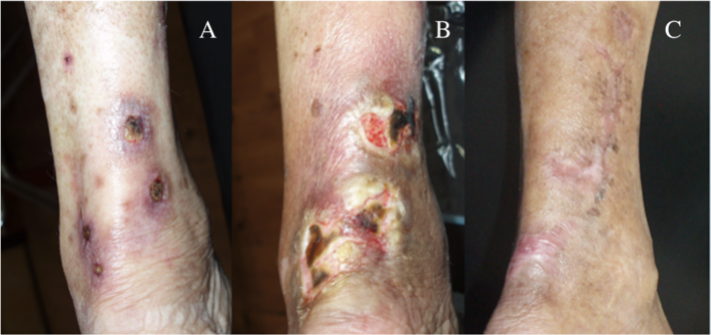
**Photographs of calciphylaxis lesions on the left calf. (A)** Six weeks after onset, the lesions became ulcerative, exhibiting surface eschars. **(B)** Before surgical debridement, the lesions showed necrotic changes as the ulcers expanded. **(C)** The lesions healed completely after six months of multimodal treatment.

**Figure 2:**
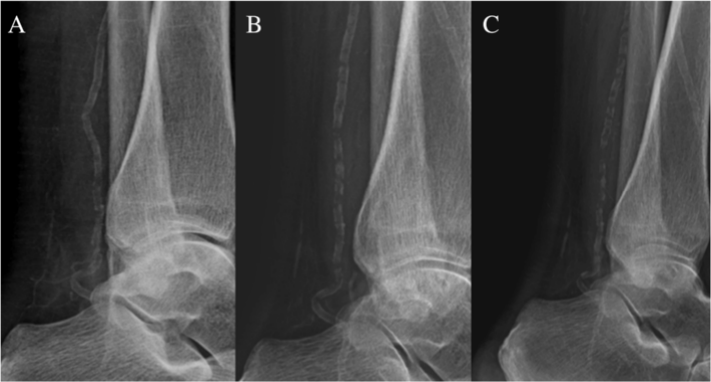
**Plain radiographs of the left lower leg and ankle. (A)** Six weeks after the onset of skin lesions, substantial calcifications were visible in the posterior tibial artery and a network of small vessels in the retromalleolar region, near the skin lesions. **(B)** After 11 months, the vessel calcification in the retromalleolar region was almost completely resolved and calcification in the posterior tibial artery showed apparent regression. **(C)** After a subsequent 11 months of cinacalcet treatment, the posterior tibial artery demonstrated further calcification regression.

**Figure 3:**
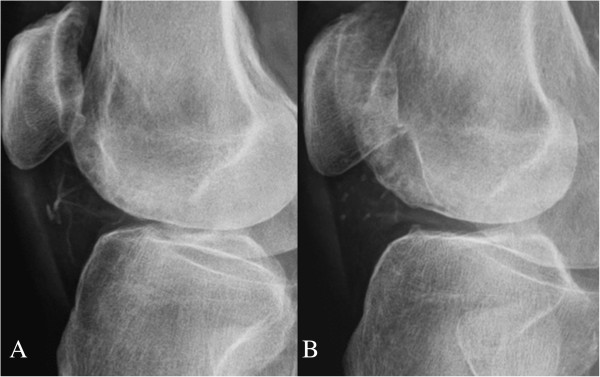
**Plain radiographs of the right knee. (A)** Before specific treatment, the joint space exhibited obvious small vessel calcification. **(B)** After 11 months, the small vessel calcification showed substantial regression.

**Figure 4:**
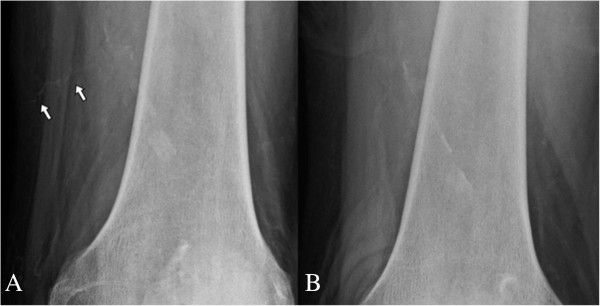
**Plain radiographs of the right thigh. (A)** A calcified subcutaneous vessel (arrows) presented before specific treatment. **(B)** The calcified small vessel was completely absent after 11 months.

**Figure 5:**
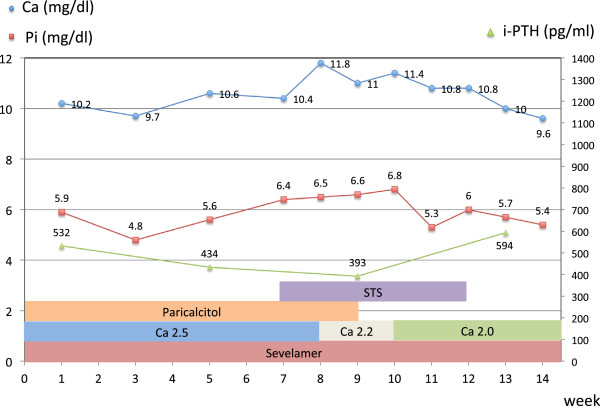
**Serum calcium, phosphate, and intact parathyroid hormone levels in the first 14 weeks of the disease (the period before surgical debridement).** The sodium thiosulfate treatment began in week 7 and ended in week 12. During this period, the predialysis calcium levels markedly increased compared with the levels before and after treatment. Paricalcitol (4μg, three times per week) was discontinued in week 9 because hypercalcemia persisted. The dialysate calcium concentration was decreased from 2.5 to 2.2mEq/L, and then to 2.0mEq/L, stepwise (presented as Ca 2.5, Ca 2.2 and Ca 2.0). Sevelamer was used throughout the course of treatment. STS, sodium thiosulfate; Pi, serum phosphate; i-PTH, intact parathyroid hormone.

In the ensuing weeks, her lesions became ulcerative and eschars covered the wound surfaces (Figure 1A). Plain radiographs indicated marked calcification in the vessels of her lower extremities (Figures 2A, 3A and 4A). A network of calcified vessels as small as 0.5mm in diameter was located in the retromalleolar region of her left leg, near the skin lesions (Figure 2A). Calciphylaxis was suspected and intravenous STS was administered three times a week after each session of hemodialysis. The maintenance dose was 12g, equivalent to 15.4g per 1.73m^2^ of body surface area. Her predialysis serum calcium levels demonstrated a notable change from 10.4mg/dL to as high as 11.8mg/dL after initiating STS therapy. The dialysate calcium concentration was subsequently decreased from 2.5 to 2.0mEq/L stepwise and dialysis frequency was increased from three to four times per week. Treatment with paricalcitol (4μg, three times per week) was also stopped. As a result, her predialysis calcium levels decreased to 10.8mg/dL during the remaining STS treatment course (Figure 5). Nevertheless, necrotic changes continued as the ulcers expanded. STS was discontinued after five weeks of treatment. The laboratory data showed that her predialysis calcium levels declined to less than 10mg/dL in one week. Despite a series of efforts including hyperbaric oxygen therapy, diligent wound care and pain control, the lesions worsened and eventually required surgical debridement (Figure 1B).

**Figure 6:**
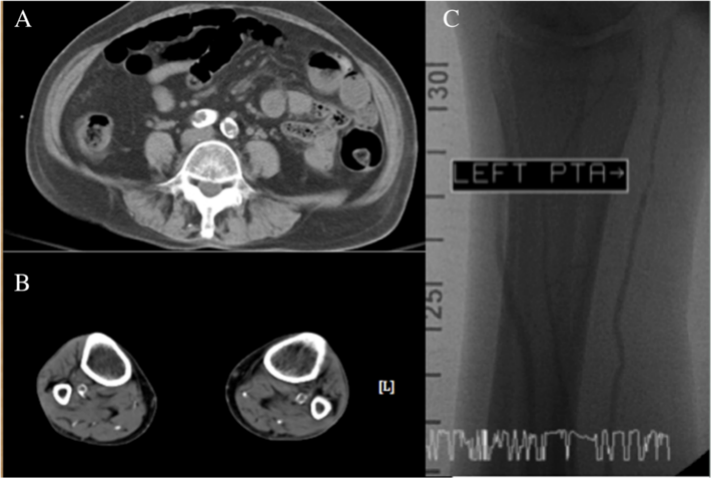
**Diffuse calcifications and atherosclerotic changes of the vessels.** Computed tomography angiographies without enhancement showed **(A)** marked vascular wall calcifications and atherosclerotic changes of bilateral common iliac arteries and **(B)** marked calcification of the large and small arteries of both lower legs. **(C)** Conventional angiography showed a 70% stenosis in left posterior tibial artery, indicating atherosclerotic change of the vessel.

Three months after the onset of skin lesions, our patient was hospitalized and scrupulous debridement was performed to excide the gangrenous tissue. The pathology of the gangrenous tissue revealed necrotic debris and inflammatory cellular exudates. The wound culture exhibited *Acinetobacter baumannii* growth, so intravenous amoxicillin-clavulanate was administered based on a sensitivity test. Because of the active infection of the skin lesions and fear of deepening or expanding the wound, no tissue biopsy was performed. Computed tomography angiography revealed diffuse vascular wall calcifications and atherosclerotic changes of the low abdominal aorta and bilateral iliac arteries, and marked calcification of the large and small arteries of both lower extremities (Figure 6A,B). Conventional angiography showed a 70% stenosis in her left posterior tibial artery (Figure 6C) and a percutaneous transluminal angioplasty was performed for dilation. Our patient was discharged after one month of hospitalization, showing improvement of the lesion after aggressive wound care. Cinacalcet was concomitantly administered to treat the hyperparathyroidism and hypercalcemia. Her predialysis calcium level declined from 9.8 to 8.2mg/dL in two weeks. We adjusted the cinacalcet dosage between 25 and 50mg/day and the dialysate calcium concentration between 2.5 and 3.0mEq/L to avoid symptomatic hypocalcemia. The lesions steadily improved and were completely healed two months after discharge (Figure 1C). Repeated plain radiographs in the following two years revealed gradual VC regression in both lower extremities (Figures 2B,C, 3B and 4B). The network of small vessel calcifications in the retromalleolar region almost completely resolved. During a two-year follow-up period, during which cinacalcet treatment continued, her serum calcium levels typically remained at approximately 8.4 to 9.4mg/dL, her phosphate at 4.1 to 6.4mg/dL, and her intact parathyroid hormone at 250 to 480pg/mL. No vitamin D analogues or calcium-containing phosphate binders were used. Furthermore, no skin lesions recurred.

## Discussion

Calciphylaxis lesions typically affect the lower limbs, but can appear on the abdomen, buttocks or breasts. According to a clinical study, the most common site of initial involvement is located 2 to 5cm above the Achilles tendon [1]. Clinical manifestations of calciphylaxis include violaceous skin lesions and subcutaneous nodules or plaques that can develop into painful ulcers, exhibiting eschars or necrosis. We made a presumptive clinical diagnosis of calciphylaxis in our patient based on her characteristic clinical presentation, contributory history, radiographic findings and exclusion of other mimicking diseases, and treated her accordingly. This case was limited by the lack of a tissue diagnosis; nevertheless, it is noteworthy for various reasons. First, we used plain radiographs as a convenient and useful method to assess the evolution of VC. Second, after using multimodal therapeutic efforts, including STS and cinacalcet, her VC gradually regressed. Third, intravenous STS yielded elevated serum calcium levels; however, this phenomenon and its clinical significance require confirmation.

A simple imaging method that is widely available, cost-effective and delivers only a small dose of radiation is required to screen for cardiovascular calcification. Mammography was postulated as superior to plain radiography and computed tomography for revealing arteriolar calcification in areas of calciphylaxis [5]; however, its utility must be validated. In one study, cardiovascular calcification screenings conducted using plain radiographs showed a strong correlation with computed tomography measurements [6]. The presence of arterial calcifications on plain radiographs has been classified into intimal calcification, exhibiting a patchy distribution, and medial calcification, exhibiting a linear or railroad-track arrangement. In a study of seven patients with calciphylaxis, the plain radiographs of all patients demonstrated linear calcification of the small vessels [2]. Plain radiographs of our patient yielded the same pattern of linear calcification over both lower limbs, including a network of small vessel calcifications in the diseased area of calciphylaxis. A recent study reported that a net-like calcification pattern demonstrated a strong association with calciphylaxis (odds ratio, 9.4) and a specificity of nearly 90% [3].

STS is used to treat calciphylaxis because it acts as an antioxidant in reversing endothelial dysfunction and binds with calcium deposits in tissues, forming a highly soluble calcium thiosulfate salt. However, in the largest case series that used a consecutive design to reduce reporting bias, only two of six STS-treated patients were complete responders [7]. Although STS treatment did not ameliorate the wound in our patient, we cannot exclude its possible contribution to the regression of VC. A study of patients on hemodialysis showed that STS could decrease the rate of VC progression [8]. Another clinical study proved that STS delayed the progression of coronary artery calcification; however, in the treatment group, the bone mineral density of the hip substantially declined [9]. We discontinued using STS early in the treatment of our patient not for a lack of clinical improvement, but because of concerns regarding the unanticipated serum calcium surge and its possible clinical effects. If the excess calcium was primarily derived from bone tissue, the pre-existing osteoporosis of our patient would be exacerbated.

No previous report has described elevated serum calcium levels when STS was used in clinical settings. In a case of peritoneal dialysis, the intraperitoneal administration of STS to treat calciphylaxis increased the calcium concentration in peritoneal effluent, leading to the removal of excess calcium [10]. Given the powerful effects of mobilizing tissue calcium, an increase in the serum calcium level seems reasonable when STS is administered intravenously. After a careful literature analysis, we determined that researchers typically made therapeutic adjustments to minimize calcium loading and optimize calcium removal when STS was administered; these adjustments might conceal the calcium-elevating effects of STS. Our clinical findings are supported by a controlled animal study in which calcium concentrations rose from the baseline concentration 45 minutes after administering STS [11].

Cinacalcet is a calcimimetic that acts on the calcium-sensing receptor of parathyroid cells, reducing parathyroid hormone and attenuating hypercalcemia. In addition to cases of healing calciphylaxis, it has been reported that cinacalcet treatment reduces vascular and pulmonary calcification [12]. In a study of uremic rats treated with cinacalcet, the bone formation rate was normalized and VC development was inhibited [13]. The concomitant reduction of VC in these animals might have resulted from the intensified mineralization of the osteoid limiting the availability of calcium and phosphate for deposition in the vascular wall. Because calcium-sensing receptors can be found on vascular smooth muscle cells, cinacalcet is believed to directly affect VC inhibition. In humans, a controlled study of patients on hemodialysis showed that cinacalcet inhibits the progress of coronary artery calcification [14]. In the ADVANCE study, patients who received cinacalcet and low-dose vitamin D demonstrated less VC progression than did patients who were administered vitamin D analogues alone [15]. To the best of our knowledge, no reports have explored how combined STS and cinacalcet therapy affects VC evolution.

It is difficult to determine the precise mechanism of multimodal treatment responsible for the regression of VC and wound healing. According to the clinical course, the necrotic wounds of our patient began healing only after surgical debridement. The percutaneous transluminal angioplasty procedure used to treat the stenosis in her left posterior tibial artery might also have contributed to the healing of the lesions. However, VC regression occurred not only in her lower left leg but also in her right thigh and knee, where no percutaneous transluminal angioplasty was performed; thus, this procedure fails to explain the effects of VC regression. Although the VC regression could have been the natural course of the disease, the tendency of VC to progress casts doubt on this possibility. Among the therapeutic modalities used to treat our patient, no single treatment could have contributed more to the VC regression than did cinacalcet and STS; various clinical studies have demonstrated the inhibitory effects of STS and cinacalcet on the progression of VC. During the two-year course of the gradual VC regression exhibited by our patient after wound healing, cinacalcet was the only specific treatment that could interfere with vessel calcification. However, based on accumulated clinical reports, VC regression is not common after cinacalcet treatment. To explain the rare and astonishing regression of VC in our patient, we suspect that the preceding STS treatment dissolved a portion of calcium precipitate in the vascular wall, changing the composition or structure of the calcification to facilitate the inhibitory effect of cinacalcet on calcification. Nevertheless, additional evidence is required to support this hypothesis.

## Conclusions

Plain radiography could be a convenient and useful method to assess the evolution of VC. Because VC is highly associated with cardiovascular mortality, we require an effective method to not only delay but reverse the progression of this highly pathogenic illness. In addition to a favorable wound outcome, our patient exhibited regressed calcifications of the large and small arteries in her lower limbs. According to the clinical course, the surgical treatment and percutaneous transluminal angioplasty procedure substantially contributed to wound healing. Regarding the regression of VC, treatment with cinacalcet and STS might have played distinct roles. A well-controlled study or large case series should be conducted to assess the additive effects of these agents in treating VC.

## Consent

Written informed consent was obtained from the patient for publication of this case report and accompanying images. A copy of the written consent is available for review by the Editor-in-Chief of this journal.

## Abbreviations

STS: sodium thiosulfate; VC: vascular calcification.

## Competing interests

The authors declare that they have no competing interests.

## Authors’ contributions

HY conducted the literature review and was the chief author of the manuscript. HY and IH were involved in the diagnosis and management of the patient. CC contributed to clinical management of the patient during the hospitalization as well as acquisition and interpretation of medical images. YH contributed to critical revision of the manuscript for important intellectual content. All authors read and approved the final manuscript.
